# CLIMB-COVID: continuous integration supporting decentralised sequencing for SARS-CoV-2 genomic surveillance

**DOI:** 10.1186/s13059-021-02395-y

**Published:** 2021-07-01

**Authors:** Samuel M. Nicholls, Radoslaw Poplawski, Matthew J. Bull, Anthony Underwood, Michael Chapman, Khalil Abu-Dahab, Ben Taylor, Rachel M. Colquhoun, Will P. M. Rowe, Ben Jackson, Verity Hill, Áine O’Toole, Sara Rey, Joel Southgate, Roberto Amato, Rich Livett, Sónia Gonçalves, Ewan M. Harrison, Sharon J. Peacock, David M. Aanensen, Andrew Rambaut, Thomas R. Connor, Nicholas J. Loman, The COVID-19 Genomics UK (COG-UK) Consortium

**Affiliations:** 1 Institute of Microbiology and Infection, University of Birmingham, Birmingham, UK; 2 Pathogen Genomics Unit, Public Health Wales NHS Trust, Cardiff, UK; 3 Centre for Genomic Pathogen Surveillance, Wellcome Genome Campus, Hinxton, UK; 4 Oxford Big Data Institute, Old Road Campus, Oxford, UK; 5 Health Data Research UK Cambridge, Wellcome Genome Campus, Hinxton, UK; 6 Institute of Evolutionary Biology, University of Edinburgh, Edinburgh, UK; 7 Wellcome Sanger Institute, Hinxton, UK; 8 Department of Medicine, University of Cambridge, Cambridge, UK; 9 Department of Public Health and Primary Care, University of Cambridge, Cambridge, UK; 10 School of Biosciences, The Sir Martin Evans Building, Cardiff University, Cardiff, UK; 11 Quadram Institute, Norwich, UK; 12 https://www.cogconsortium.uk

## Abstract

In response to the ongoing SARS-CoV-2 pandemic in the UK, the COVID-19 Genomics UK (COG-UK) consortium was formed to rapidly sequence SARS-CoV-2 genomes as part of a national-scale genomic surveillance strategy. The network consists of universities, academic institutes, regional sequencing centres and the four UK Public Health Agencies. We describe the development and deployment of CLIMB-COVID, an encompassing digital infrastructure to address the challenge of collecting and integrating both genomic sequencing data and sample-associated metadata produced across the COG-UK network.

**Supplementary Information:**

The online version contains supplementary material available at 10.1186/s13059-021-02395-y.

## Introduction

Combining genomic sequencing of pathogens with epidemiology as part of a response to an outbreak has demonstrated success in epidemiological investigations of viruses such as Ebola, Yellow Fever and Zika [1]. Pathogen genomes are useful for reconstructing a phylogenetic history of an outbreak and are now being used in real-time to assist epidemic response.

Established sequencing networks already exist for some infectious pathogens. As an example, the GenomeTrakr Network is part of the US Food and Drug Administration and connects labs across the USA and internationally to sequence foodborne bacterial pathogens and since 2013 the project has sequenced nearly 500,000 isolates. Flu viruses are also routinely sequenced, both through the use of Sanger and whole genome sequencing (WGS) techniques. Public health agencies in the UK operate seasonal influenza surveillance programmes using WGS, with results reported to both governments and international organisations such as the WHO and ECDC. While genomic data is increasingly used within public health agencies for retrospective surveillance activities, the benefits of genomic epidemiology are yet to be fully realised for prospective and proactive outbreak response. This is exemplified in the current pandemic, where the initiation of programmes for sequencing of SARS-CoV-2 typically lagged behind planning for other parts of the pandemic response. The utility of genomic data has been such that this should be the last pandemic where genomic epidemiology is not a core part of pandemic planning.

Most existing public health sequencing initiatives are built around whole genome sequencing capacity afforded by facilities in large hospitals and public laboratories. However, with the emergence of lower capital cost sequencing instruments such as Oxford Nanopore platforms, genomic sequencing is now available to smaller regional hospitals and academic laboratories, vastly expanding the sequencing capacity for a hypothetical surveillance network. Such technology is small and cost-effective enough to conduct sequencing of small pathogen genomes in the field, in the clinic and in the classroom. However, with this democratisation of sequencing technologies, a new challenge emerges in how data generated across many different laboratories can be collated, compared and analysed to support outbreak/pandemic response simultaneously at local, regional, national and global levels.

The COVID-19 Genomics UK (COG-UK) consortium was established in March 2020 with the aim to deliver large-scale and rapid whole-genome virus sequencing and analyse the sequences for local NHS centres and the UK government [2]. COG-UK is a national partnership of NHS organisations, the four UK Public Health Agencies, the Wellcome Sanger Institute and over 20 academic partners. The work of the consortium generates reports for the UK Scientific Advisory Group for Emergencies (SAGE), as well as providing analyses and advice to the UK devolved administrations. This is the first time that genomic epidemiology has been used at a national scale to guide a response to a pandemic in the UK, as demonstrated in regular reports to the UK’s Scientific Advisory Group for Epidemics (https://www.cogconsortium.uk/news-reports/sage-reports/).

**Fig. 1:**
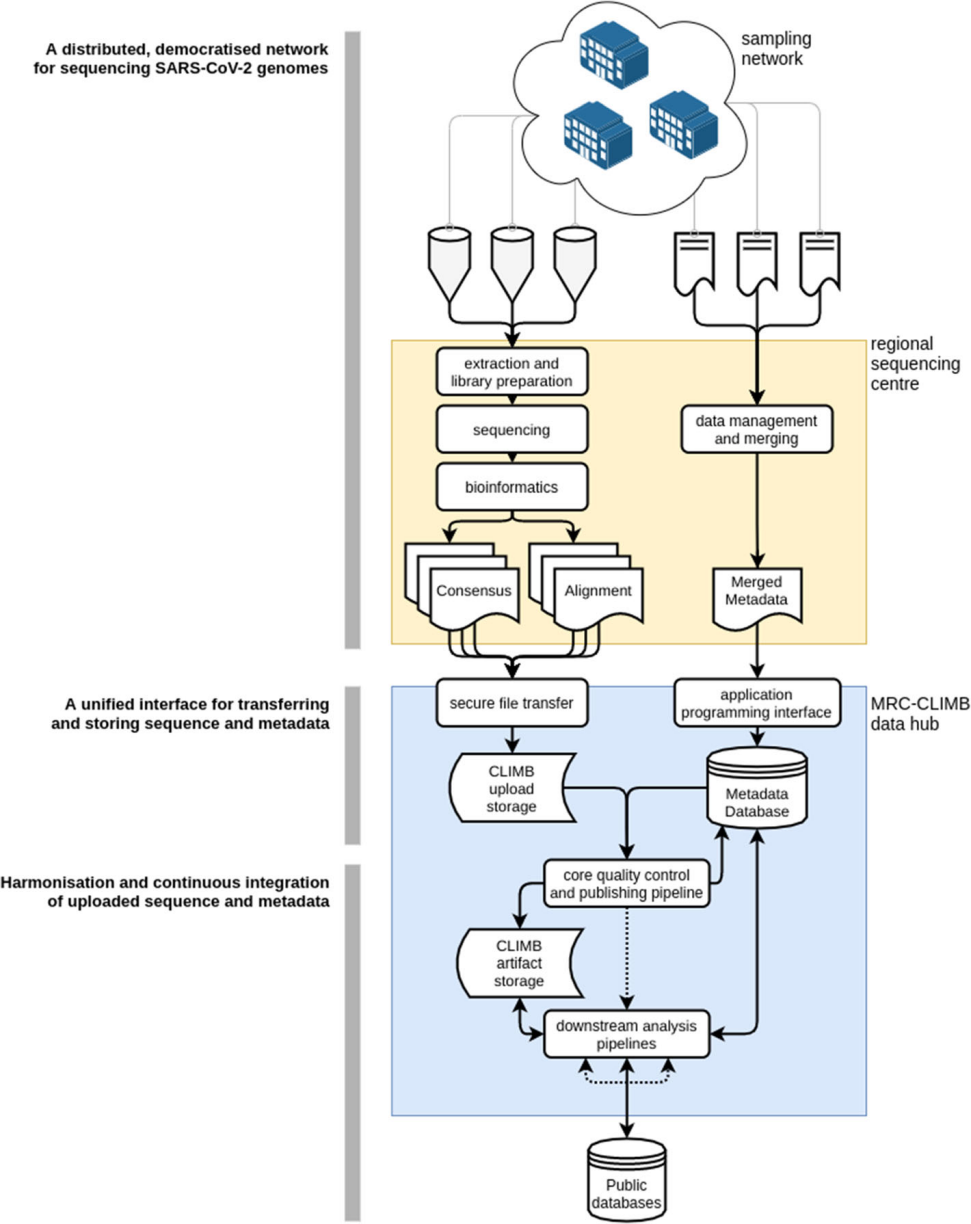
Overview of the COG-UK data flow. (Top) A network of sampling sites (e.g. hospitals and testing centres) produce samples and sample metadata which are received by a regional sequencing centre. The sample is extracted and sequenced and a locally run bioinformatics pipeline generates both a consensus viral genome and an alignment of sequenced read fragments against the SARS-CoV-2 reference genome. (Middle) The consensus sequence and alignments are uploaded via secure file transfer to be stored on CLIMB-COVID. Metadata is securely transferred over HTTPS to an application programming interface (API) that transforms metadata into a model to be stored in a database on CLIMB-COVID. (Bottom) The core quality control pipeline executes every day to integrate newly uploaded samples and metadata into the single canonical dataset of all uploaded sequences. Once this pipeline is finished, it notifies downstream analysis pipelines through a messaging protocol to generate analysis artifacts like phylogenetic trees. Downstream analysis pipelines also automatically deposit genomes in public databases such as GISAID and ENA/INSDC

As well as rapidly responding to the problems of how to extract and sequence SARS-CoV-2 genomes, another key challenge for COG-UK was to develop an infrastructure capable of harmonising data from a network of sources to create one dataset for analysis. The development of this system posed many interesting and challenging problems from a technical standpoint. In this article, we present several of these problems, our solutions and what we have learned from the process. Our system provides a model (Fig. 1) that may serve as a foundation to inform others who are faced with the challenge of designing and deploying a similar system to aid outbreak tracking in this or future pandemics.

## Results

We present a model of our system (Fig. 1), which can be broken down into three core functions:

- Produce data, by connecting a network of regional sequencing sites (academic or government affiliated) to a network of sampling organisations, to establish a distributed, democratised network for sequencing SARS-CoV-2 genomes
- Collect data by providing a system to transfer sequencing data, consensus genomes and sample metadata that works in the same way for every member of the consortium
- Integrate data into a single dataset by harmonising the collected sequences and metadata

### An autonomous and scalable network for decentralised sequencing of SARS-CoV-2 genomes

The COG-UK consortium forms a national network of organisations that in combination collect and sequence samples. The organisations within the consortium have a high degree of autonomy. This autonomy is valuable as sites can take advantage of their own local expertise to make decisions on protocols and methods to use for sample collection, preparation and sequencing, reducing the burden for an organisation that wishes to participate. Some of these sampling sites have the capacity and resources to perform their own sequencing, those that do not are connected to a regional sequencing organisation, or the Wellcome Sanger Institute (WSI). Regional sequencing sites include academic institutions, small laboratories and public health agencies. Connecting sampling organisations to a local sequencing laboratory means sequenced genomes can be turned around within 24–48 h of sample collection.

**Fig. 2:**
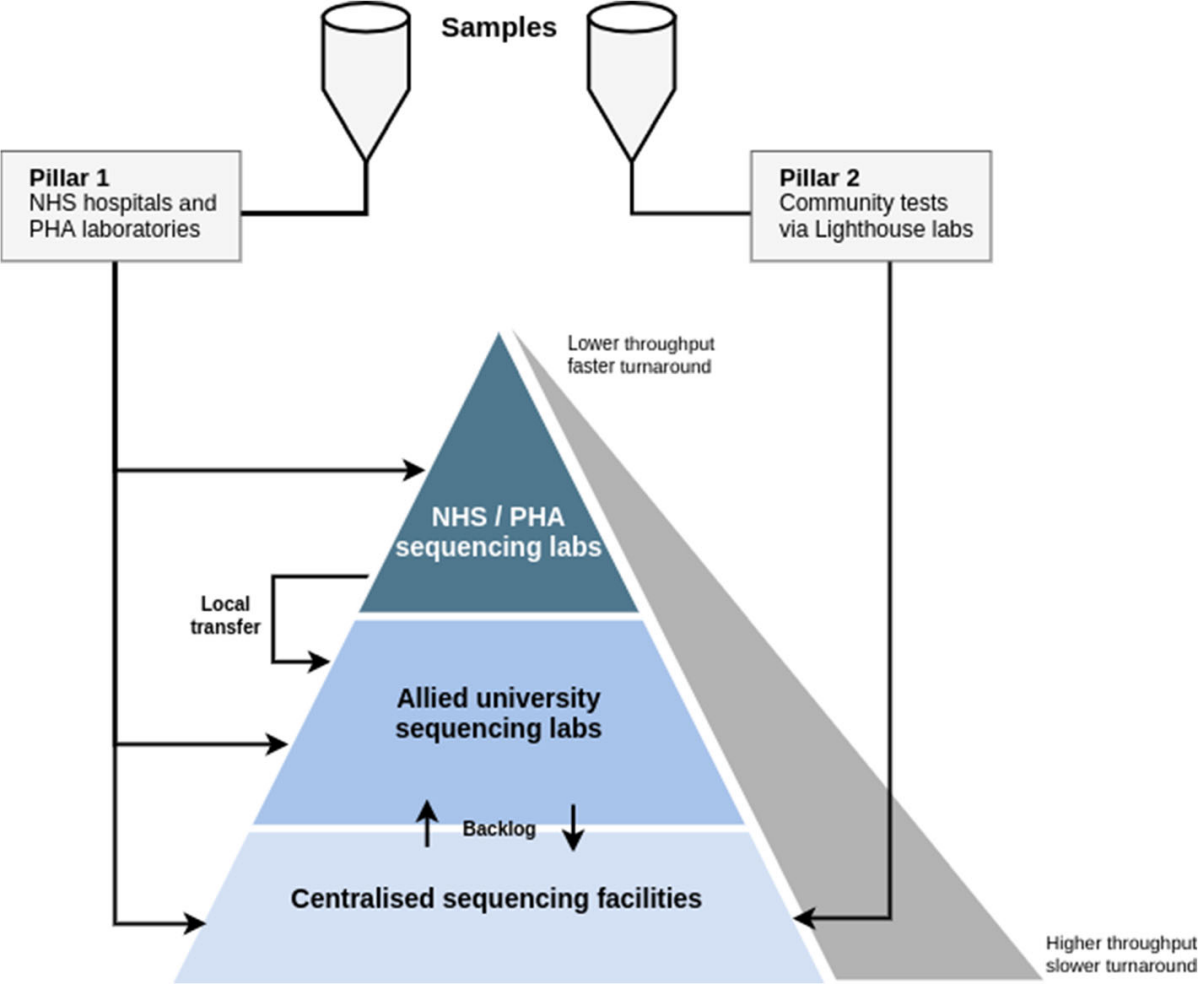
COG-UK sequencing model. Samples are sourced from two “pillars”; pillar 1 samples are collected across the NHS and Public Health Agencies, pillar 2 samples are collected at the Lighthouse Labs at particular strategic sites in the UK. Generally, pillar 1 samples are received by NHS labs who process them for sequencing locally or by a university sequencing lab for a fast turnaround. Pillar 2 samples are generally shipped through to the Wellcome Sanger Institute for high-capacity sequencing

This two-tiered sequencing model has facilitated both a prioritised, rapid regional response, as well as supporting lower priority, high-throughput projects such as the sequencing of every positive sample from the Lighthouse Laboratories (Fig. 2).

However, this autonomy comes at a cost: raising the difficult challenge of coordinating such a diverse network of sites, using a spectrum of methods for sample extraction, PCR, library preparation, sequencing and consensus-generating bioinformatics. The core problem we faced when tasked to build this infrastructure is one of data interoperability. With geographically dispersed sequencing operations and the four public agencies all producing data with a wide variety of different techniques and platforms, it was necessary to deploy an infrastructure to collate this data into a single, consistent, canonical data set, available for everyone within the consortium and for consistent public dissemination.

#### A hub model for integrating genomic and epidemiological data

We chose to form a hub model around the Cloud Infrastructure for Microbial Bioinformatics (CLIMB) compute facility [3]. CLIMB is not just a pragmatic choice given the affiliation of the authors; since it was first deployed in 2014, it has provided infrastructure to microbiologists to produce and use software for the analysis of genomic data sets, serving over 300 research groups at more than 85 organisations spread across the UK. It was designed as a system to support microbial bioinformatics and has been used for pathogen outbreak analysis in the past [4].

We formed CLIMB-COVID as a ‘walled garden’ within existing MRC-CLIMB infrastructure, for the purpose of providing a central, replicated environment for the storage and analysis of data generated by COG-UK. CLIMB served as a trusted research environment with no affiliation to any one country or public health agency, enabling cooperation across a diverse network of sequencing operations covering four countries, and the development of a bespoke service and environment to meet the needs of the project.

Sites participating in the consortium maintain authority over the data they generate, interpreting and sharing it to inform a local public health response. As part of their membership, they are responsible for transferring the sequenced consensus FASTA file, and an alignment of the sequenced reads against the SARS-CoV-2 reference genome [5] as a BAM to a designated server. This simplifies analysis within CLIMB-COVID, and also enables the hub to avoid storing human reads sequenced incidentally as part of SARS-CoV-2 sequencing, while also providing valuable data that can be used to perform additional analyses for scientific or quality control purposes.

To assist with the on-boarding of new sites, including those with limited bioinformatics support we also built a reproducible Nextflow pipeline (https://github.com/connor-lab/ncov2019-artic-nf) that enables the processing of data for sites following the ARTIC sequencing protocols [6].

#### A walled garden for fast turn around and to maintain sequence integrity

This hub model operates with a different paradigm to one suggested recently by Black et al. [7], which recommended that raw reads would first be uploaded to the SRA, or Illumina BaseSpace, and that the final step of any assembly pipeline would be automatic submission to one of the International Nucleotide Sequence Database Collaboration (INSDC) databases, or a pathogen specific initiative such as GISAID. In practice, this paradigm would introduce unnecessary delays in the processing of data and hamper real-time genomic surveillance efforts. In building our system, the focus has been on generating actionable information to support public health action as rapidly as possible.

Our approach instead takes sequence data for initial analysis inside a system hosted on MRC-CLIMB which can only be accessed by members of the consortium. This ensures the data is immediately usable, as sequences can be transferred to the consortium as soon as they have been processed locally, whereas large public databases often have a lead time up to a few days before accessions are indexed and resources can be downloaded, which is incompatible with the goal to turn around sequences within 24 h.

Our model also allows our internal pipelines to be tolerant of the different error profiles we may expect to see given the diverse sequencing methodologies in use across the sites. Processing data centrally allows us to perform basic quality control and ensure consensus genomes are internally consistent before they are distributed outside the consortium, mitigating the risk of polluting international databases. Sequences are only processed and integrated into the data set if they have been uploaded to CLIMB-COVID, which enforces an environment that fosters data sharing. Consortium members additionally benefit from sharing data via CLIMB-COVID, as we manage automatically uploading data to public databases on their behalf.

#### A minimal metadata standard to ensure wide adoption of data collection

For the sequenced genomes to be useful, it is essential to pair them with metadata that contextualises the time, place and circumstance of the collected sample. This context is what allows us to use genomic epidemiology to drive an effective intervention as part of a public health response.

There are already several well defined lists of metadata that are recommended for collection, for example submissions to the European Nucleotide Archive suggest following the ‘ENA virus pathogen reporting standard checklist’ (ERC000033), and recently, the Public Health Alliance for Genomic Epidemiology (PHA4GE) drafted a specification for sharing contextual data about SARS-CoV-2 genomes to advocate the openness and reusability of generated data sets [8]. Although it is straightforward to construct a list of desired pieces of metadata to collect, the real problem is reconciling such a standard with the reality of how data can be collected on the ground. We defined a very small set of mandatory fields (Table 1) that aimed to limit the burden on laboratories (for a full table of fields refer to Table 3).

**Table 1:** COG-UK minimal mandatory metadata specification

Data item	Field name	Description	Base access level (public, consortium or restricted)	Mandatory
**Central sample ID**	central_sample_id	A unique identifier to refer to the sample within the consortium	Public	Yes
**Date of sample (collected)**	collection_date	The date the sample was collected	Public	Yes (otherwise received_date)
**Date of sample (received)**	received_date	The earliest date that this sample was known to be checked in to a diagnostic or sequencing laboratory	Public	No (unless collection_date is not provided)
**Country code**	adm1	The country in which the sample was collected	Public	Yes
**County**	adm2	The county within the UK in which the sample was collected	Consortium	Strongly recommended
**Sampling strategy**	is_surveillance	Whether this sample was collected as part of a random surveillance strategy, or a targeted outbreak analysis	Consortium	Yes

In practice, we found a sample’s identifier within the healthcare system could not be shared.

Samples are relabelled with a central sample ID (or ‘COG ID’) which identifies a sample in the consortium and in public databases. COG-UK made pre-printed barcodes available which are used by many collection sites, but are not mandatory.

As the expertise of the analyst groups within COG-UK is focused on viral phylodynamics, which looks to map the evolution of sequenced viral genomes over time, our mandatory fields are concerned with linking the date a sample was collected and the approximate geographical location it was collected in. Initially, collection county was a necessary but unfortunate compromise as the security assessments and contractual arrangements to collect and store more fine-scale location information such as outer postcode would take some time to organise.

Each metadata field has one of three access control levels: public, consortium and restricted. Public fields are highly portable and can be deposited in databases. Consortium level data must be analysed inside CLIMB-COVID or a public health agency. Access to restricted data requires a specific agreement governing the exchange and use of the metadata to be drafted.

### A unified interface for transferring and storing sequences and sample metadata

#### Centrally managing consortium data through application programming interfaces (APIs) and Majora

We centralised the storage of metadata with a bespoke software application to provide a consistent platform for validating and disseminating sample metadata. Majora (https://github.com/SamStudio8/majora/) is the database that backs the CLIMB-COVID digital infrastructure. It stores information about samples and files, referred to as ‘artifacts’. Majora also concerns itself with storing information on the ‘processes’ that have been applied to artifacts. For example, a group of sample artifacts may be pooled to form a library; a library is sequenced to provide signal data. Bioinformatics pipelines convert signal to reads, and reads to consensus genomes, and so on. Metadata is stored in one of three tiers within Majora (Table 2), based on indexing and query performance requirements. By storing a record of how each artifact comes into being, and how artifacts are linked together through processes, it is possible to build a full audit trail from when a sample was collected to any files and analyses generated about it downstream.

**Table 2:** Three tiers of metadata within Majora

Tier	Implementation	Properties	Example
Primary	Database model	● Fast queries via object-relational mapping ● Takes up space in database even if unused ● Significant work to add to the database model, API and user templates	● Biosample identifier ● Patient sex, age ● Digital resource file path, size, hash
Secondary	Database model	● Fast queries via object-relational mapping ● Additional lookups necessary to link back to the primary database model ● Cannot assume a primary model will have a secondary	● Cycle threshold metrics for biosamples ● BAM coverage metrics ● Patient healthcare worker or care home status
Tertiary	Key-value row in generic model	● More difficult to manage artifacts based on tagged properties alone ● Highly flexible ● No work required to add new tags at any time	● Locally relevant tags not implemented in a model ● Additional anonymised patient information ● Additional sequencing run information

Majora stores submitted metadata about artifacts and processes in an SQL database. Metadata is stored differently based on its priority. Fields that are a core part of a model (for example, a sample identifier, or the name of a file) are considered primary metadata and are stored in a distinct database model. Metrics such as the results of a PCR Ct test, or the coverage levels of a BAM are also stored in a distinct database model and are attached to primary models through a database foreign key. Arbitrary metadata can then be stored in key value pairs (not backed by any particular database model) and tagged to primary and secondary models as appropriate

We architected Majora as a web application so it could be easily accessed by any consortium member, and developed a collection of application programming interfaces (APIs) to avoid any human intervention delaying the validating, processing or querying of metadata. An API allows a computer programme to interface with a human or other computers. Metadata is submitted and queried by exchanging messages with Majora’s API endpoints.

**Fig. 3:**
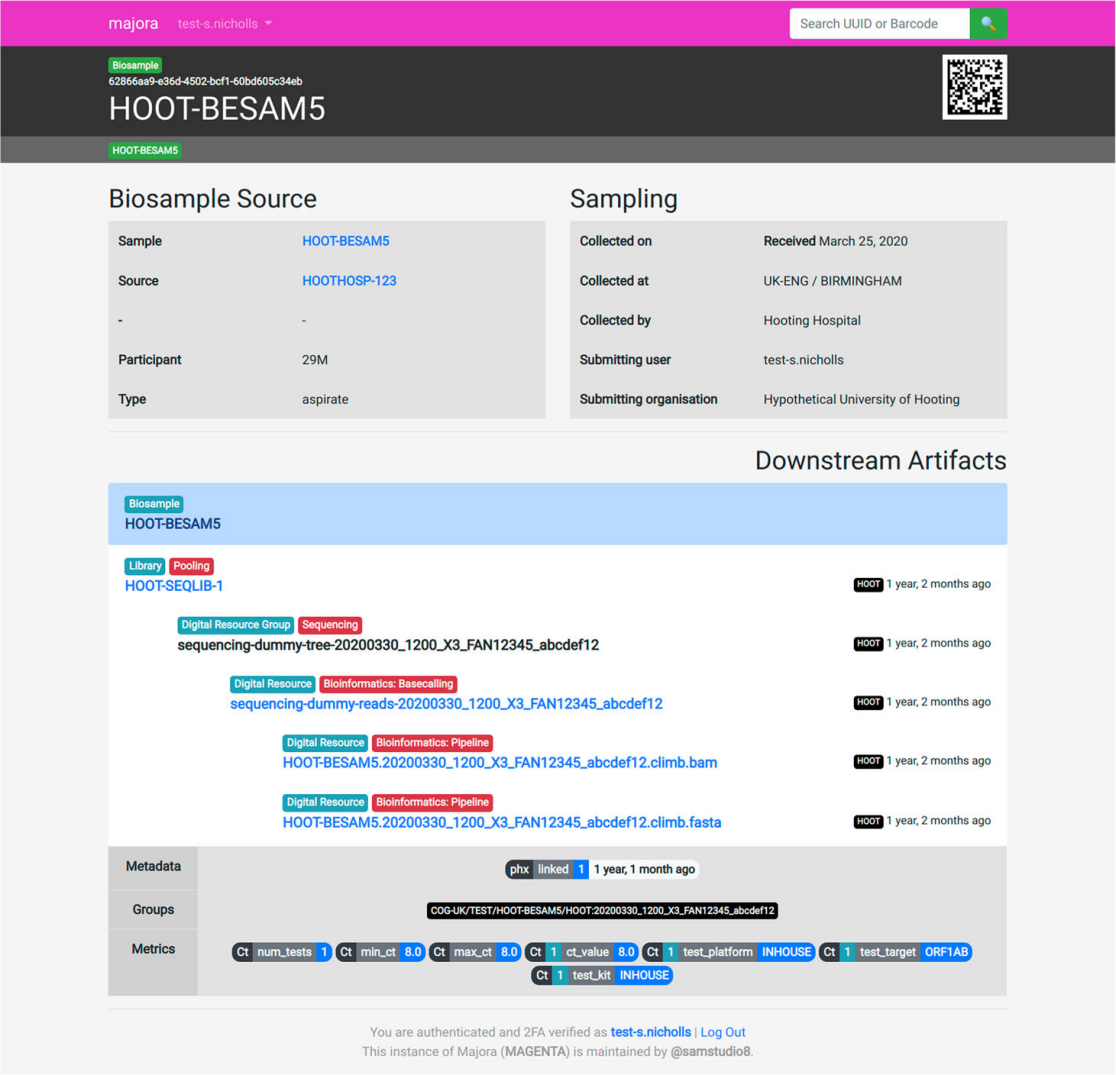
Majora web application biosample view. An example of the web interface presented by Majora to detail a biosample artifact. The downstream artifacts section allows users to see what processes have been applied to the biosample. In this example, the sample was incorporated onto a pooled sequencing library, which was sequenced and basecalled. A downstream bioinformatics pipeline resulted in a FASTA and BAM. Artifacts can be tagged with metadata and metrics. In this example, the artifact is tagged with a linkage flag and information about the sample's cycle threshold value

Majora is developed with the Django framework [9] and includes the APIs, a database of bespoke models, and a web application. The website allows for easy access to limited metadata and shows the history of processes that are known about a sample (Fig. 3). For savvy users, bots and pipelines, a command line client (Ocarina, https://github.com/SamStudio8/ocarina) has been developed that uses the API to access more advanced functionality and automate elements of metadata submission and retrieval. Advanced users can also use the API documentation to author clients of their own.

The web interface is protected by enforcing two-factor authentication on users who wish to view any metadata. The APIs are secured with a rotating key scheme that allows external applications to perform actions as a user, without the user having to provide their account password. Newer endpoints use a more straightforward, industry-standard protocol for authorization (OAuth 2.0).

As Majora is the only interface a user has to the metadata stored by the consortium, and that access is completely under our control, we can satisfy requirements set out by the NHS Digital Data Security and Protection Toolkit (https://www.dsptoolkit.nhs.uk/), enabling us to store some restricted data. For example, users are able to upload the sample identifier as it is referred to inside of the collecting site (which is considered to be restricted). These restricted identifiers are hidden from consortium users, but through Majora, users can sign an agreement that grants permission for the identifiers they have uploaded to be shared specifically with public health agencies, allowing COG-UK sequences to be linked to wider health informatics data. This has allowed the majority of samples to be linked to records held by public health agencies, who can provide supplementary metadata to COG and use the genomes in their own analyses. This layer between the users and the database where metadata is stored allows us to maintain an audit trail of who performed what actions both on the website, and through the API.

Most public and consortium level metadata can be viewed through the Majora web interfaces and API. Rather than granting a user permission to a particular access level, or deploying a cumbersome case-by-case field-level permission system, we control access to metadata by predefining a set of named views that explicitly show a subset of the metadata fields. The view itself then acts as a permission, with users making a case for why they should be granted permission to that view.

Majora allows filters to be dynamically applied to the data view to produce derivative data sets. For example, the mechanism through which we share restricted local identifiers to public health agencies will filter the samples to ensure each agency can only see samples from their own country and that the uploading user has agreed those identifiers can be shared.

#### A unified user-friendly method for uploading and validating metadata

Metadata is collected using a CSV template containing all the fields from our metadata specification (Table 3). CSV files are convenient as spreadsheet software is commonly available and intuitive to a wide range of users.

**Fig. 4:**
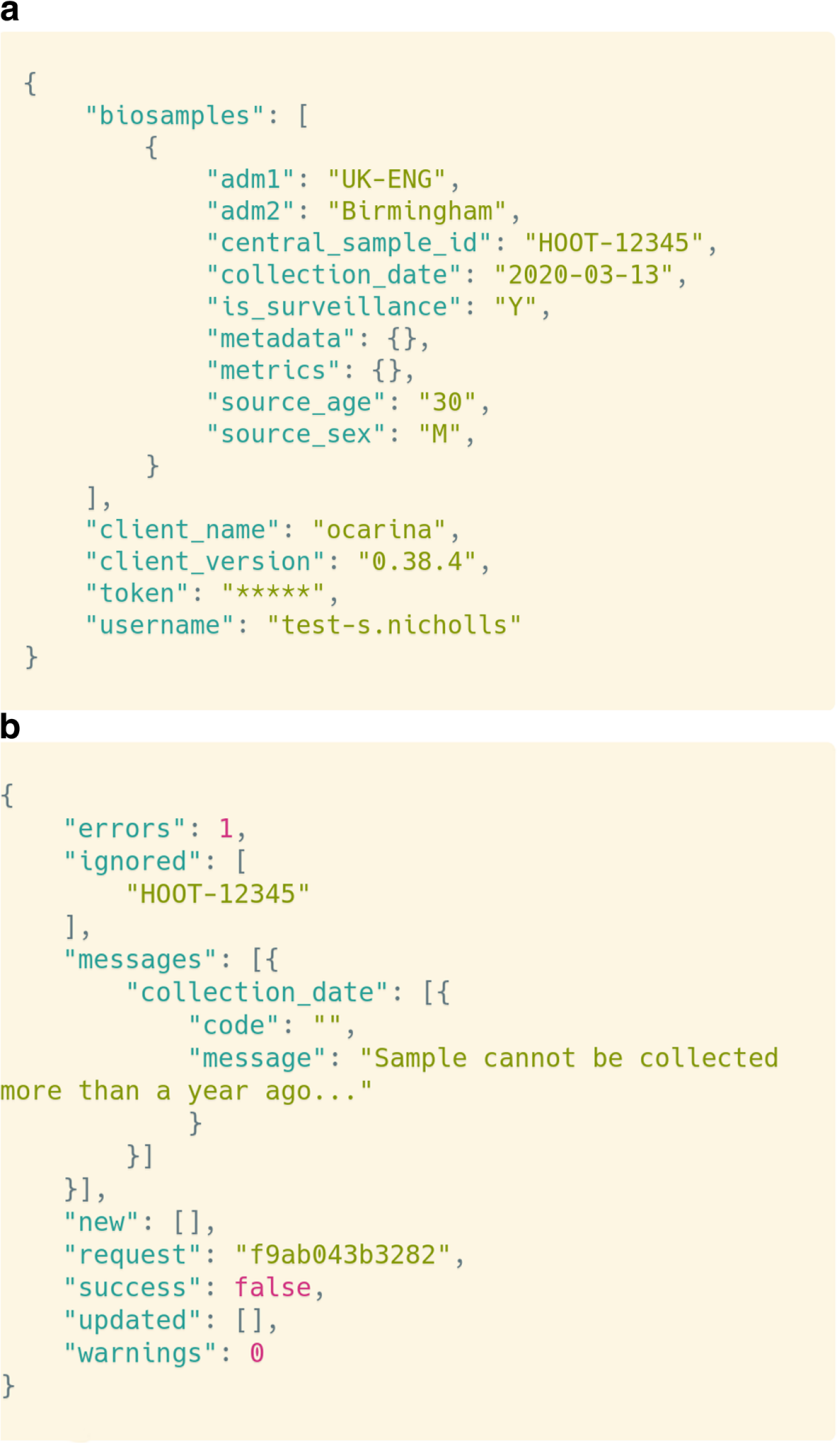
Example API request to submit a new biosample artifact to Majora. All metadata from biological samples, to library pooling processes and sequencing runs are communicated to Majora through the various API endpoints. These interfaces take structured data in the JSON format and process them to be stored in the Majora’s SQL database. This example demonstrates a simplified request to add a new biosample to Majora (**a**) and a reply from Majora indicating a validation error (**b**). Examples rendered with @carbon_app

The APIs for adding metadata to Majora require the fields to be arranged in a structured text format called JSON (JavaScript Object Notation) (Fig. 4). Messages and validation errors are returned to the API user in the same format. Although JSON can be viewed in basic text readers, or pretty printed on a command line, it is not intended for human consumption. To convert the metadata CSV files to records in Majora, a lightweight Javascript-based (Nuxt) web frontend was developed.

**Fig. 5:**
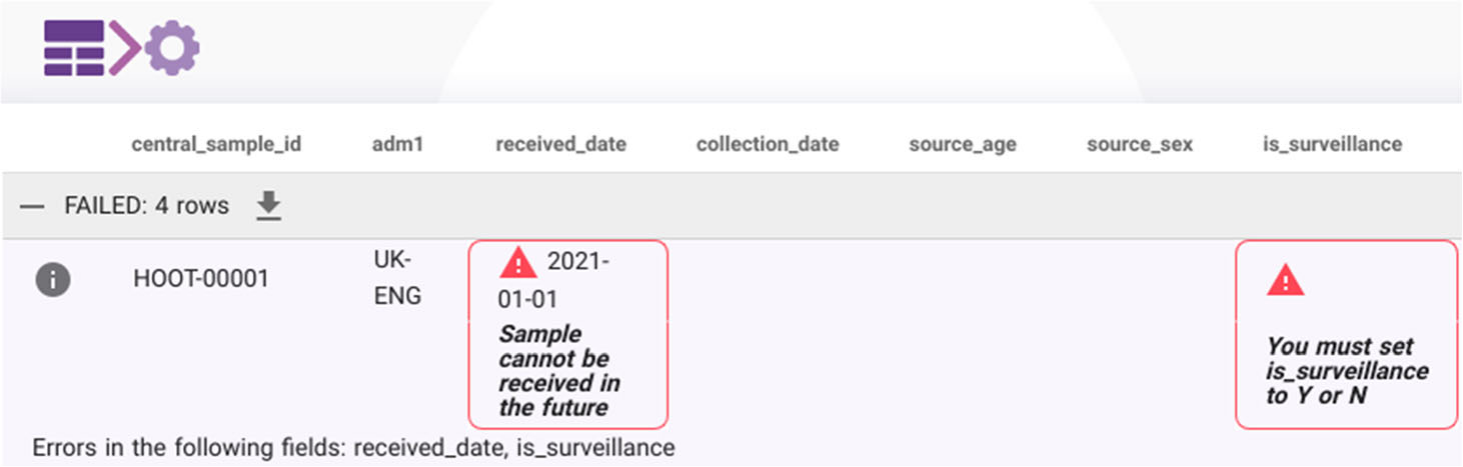
Screenshot of the metadata uploader demonstrating user-facing errors. Metadata is submitted to the consortium by uploading a filled in CSV template to the metadata uploader web application. The uploader converts the CSV data into JSON and communicates with the Majora API. Validation errors are immediately returned, parsed and displayed to the user as shown here

Users log in to the uploader with their Majora credentials and can upload their filled out metadata CSV. Data is transferred securely as the Majora API only supports secure HTTP (https). Majora’s JSON response describes any validation errors that require the user’s attention, and these are parsed and presented prominently in the uploader web application (Fig. 5). Invalid metadata is rejected by Majora and users are immediately aware of problems that must be addressed before successful submission. Valid metadata is added to the database immediately and can be queried by any other member of the consortium with access to Majora.

### Harmonisation and continuous integration of uploaded sequence and metadata

#### Elan: autonomous, scalable, daily data integration of sequences and metadata

**Fig. 6:**
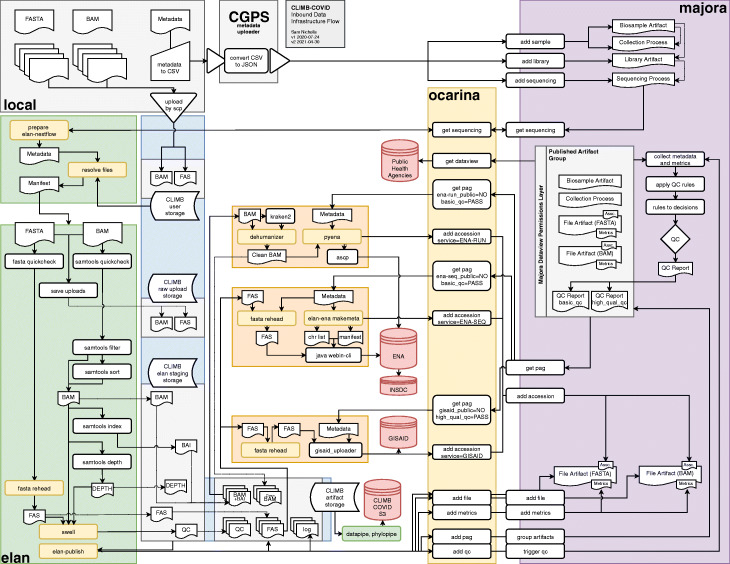
CLIMB-COVID inbound data architecture diagram. Local sites (grey, top left) generate consensus FASTA and alignment BAM for each sequenced sample. Corresponding metadata is collected and managed into a CSV using the consortium template. FASTA and BAM are uploaded to CLIMB using scp or rsync. Metadata is converted from CSV to JSON by the metadata uploader tool and passed to the Majora API to be processed (purple, right). The Elan inbound pipeline (green, left) queries the Majora metadata database using the Ocarina command line client (yellow, right). Elan matches Majora metadata to uploaded files on CLIMB (blue, left) and conducts quality control. Quality metrics are passed to Majora through Ocarina. Outbound distribution pipelines (orange, centre) are able to query Majora using Ocarina and package high-quality sequences for GISAID. ENA and INSDC databases (red). Downstream pipelines (green, centre) such as Datapipe and Phylopipe generate alignments, trees and other analysis artifacts that are shared within the consortium and made publicly available via CLIMB-COVID’s S3 storage (red). Programmes in yellow boxes indicate software built specifically for CLIMB-COVID

FASTA and BAM files uploaded to CLIMB are paired to metadata stored in Majora through a daily automated process. Unprocessed samples are flagged to be pulled into Elan, the inbound distribution pipeline. Elan (https://github.com/SamStudio8/elan-nextflow/) is an open-source pipeline built with the NextFlow workflow language [10]. Elan checks the integrity of uploaded files, calculates metrics and quality information and copies the files to an organised read-only location for downstream dissemination. Elan updates Majora about new samples that have been processed and registers their corresponding file artifacts using the APIs (Fig. 6).

**Fig. 7:**
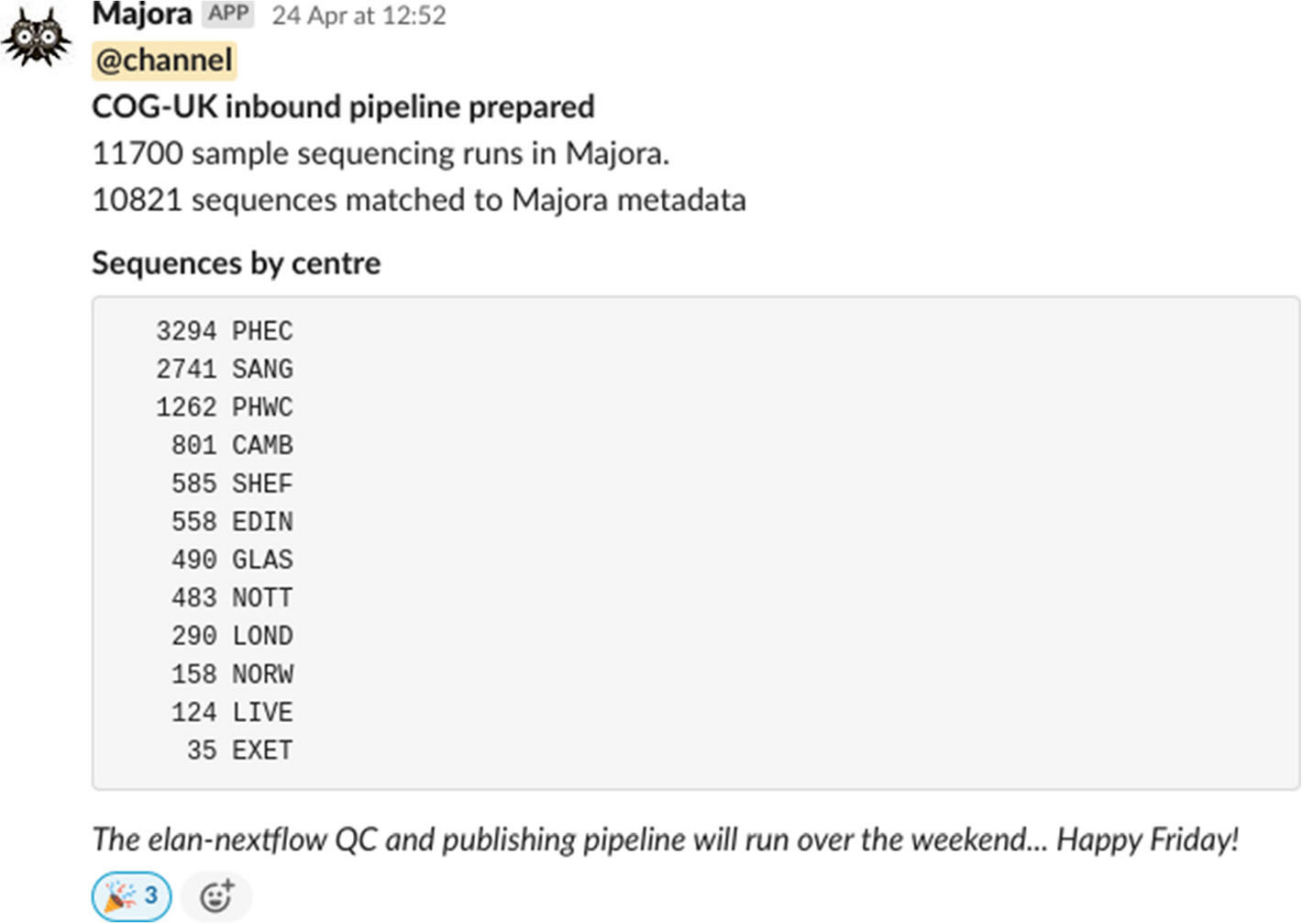
An automated Slack message announcing the start of Elan pipeline. The Elan inbound distribution pipeline is operated transparently by providing a series of courtesy messages before and after it has run. Slack messages are sent programmatically through a web hook to announce samples that appear to be missing metadata or a genome sequence. This example, dated April 24, 2020, announces that Elan was about to process the 10,000th sample

Elan is a central component of the COG-UK digital infrastructure (Fig. 7). The Elan pipeline is run every day and weekly reports are written based on data submitted by Friday, providing a natural cut-off for consortium members to aim to upload their metadata and sequences by.

#### Orchestrating data flows with human or machine readable messages

Automated announcements are sent to a well-populated Slack channel for COG-UK members responsible for collating metadata and sequence data to be alerted to missing metadata or files that should be addressed before Elan begins. When Elan has finished daily processing, an announcement counting the number of new and cumulative sequences that have passed QC is broadcast (e.g. Fig. 7).

**Fig. 8:**
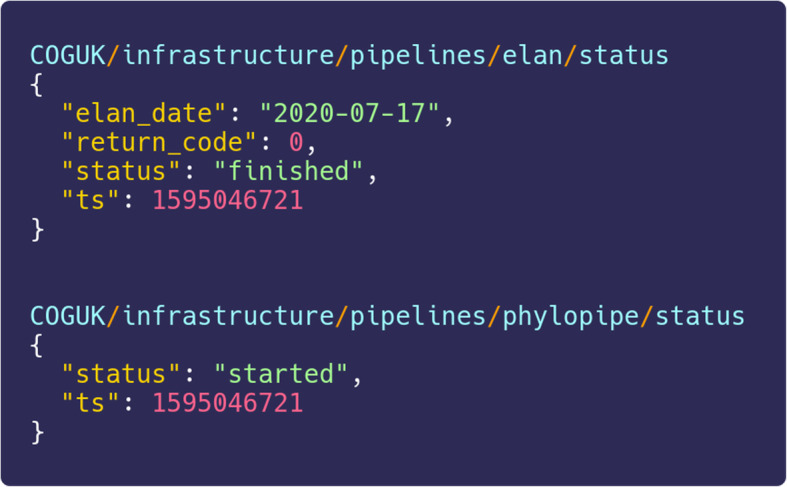
Automated machine-readable messages exchanged between pipelines from a messaging queue. To assist orchestration of pipelines, we run a message broker service that allows different pipelines within COG-UK to send messages and interact with each other. This example shows Elan’s first message on July 17, 2020, emitted to announce it has successfully completed, and the phylogenetics pipeline responding to say it has started as a result of the new data to be processed

We also deployed a Mosquitto server [11] to transmit MQTT (Message Queuing Telemetry Transport) messages between pipelines. Elan emits machine-readable messages (Fig. 8) to notify downstream pipelines that there are new samples to process. Using machine-readable messages to control other pipelines reduces human workload and encourages the development of multiple pipelines that do their particular tasks well, rather than tasks being rolled into one monolithic pipeline.

#### A QC-aware platform for querying sequences

Majora can be loaded with configurations that specify how quality control decisions should be made based on the values of uploaded metadata and metrics. For example, a mean coverage rule would specify thresholds required for a sequence to be marked as pass, warning or failure. These basic rules are building blocks grouped together by the configuration to form quality control tests. Tests can be applied (or not) based on metadata stored in Majora. For example, each sequencing platform in the consortium has its own set of rules which are conditionally applied to samples based on the platform specified in the sequencing metadata. Elan uses an API endpoint to request Majora carry out a particular QC test and store the report.

We routinely run two QC tests: basic QC is a highly tolerant test which must be passed in order for a sequence to be made available to downstream pipelines within the consortium; high-quality QC has a much stricter threshold and was initially used to determine whether samples would be shared in public databases. As Majora stores these QC results, the API endpoints that retrieve data can filter for samples that have passed (or failed) a particular QC test. Majora is able to handle QC results from different platform tests with equivalence, meaning that ‘basic QC’ for Illumina data can have different rules and thresholds when compared to Oxford Nanopore data; but users need not know which test was applied when requesting data that passes or fails basic QC.

#### Routine alignment and phylogenetic analysis of the unified data set

Datapipe, a variant calling and alignment pipeline (https://github.com/COG-UK/datapipe), is initiated by a machine-readable from Elan. Datapipe is a Nextflow [10] pipeline that combines a downsampled set of non-UK SARS-CoV-2 sequences from GISAID with the complete set of COG-UK sequences that have passed basic quality control. It applies more stringent sequence quality and metadata filtering, adds PANGO lineage assignments [12], conducts a multiple sequence alignment and calls variants. The MSA and curated metadata are published daily within the consortium and artifacts (with sensitive data removed) are made publicly available via CLIMB-COVID’s S3 object store.

**Fig. 9:**
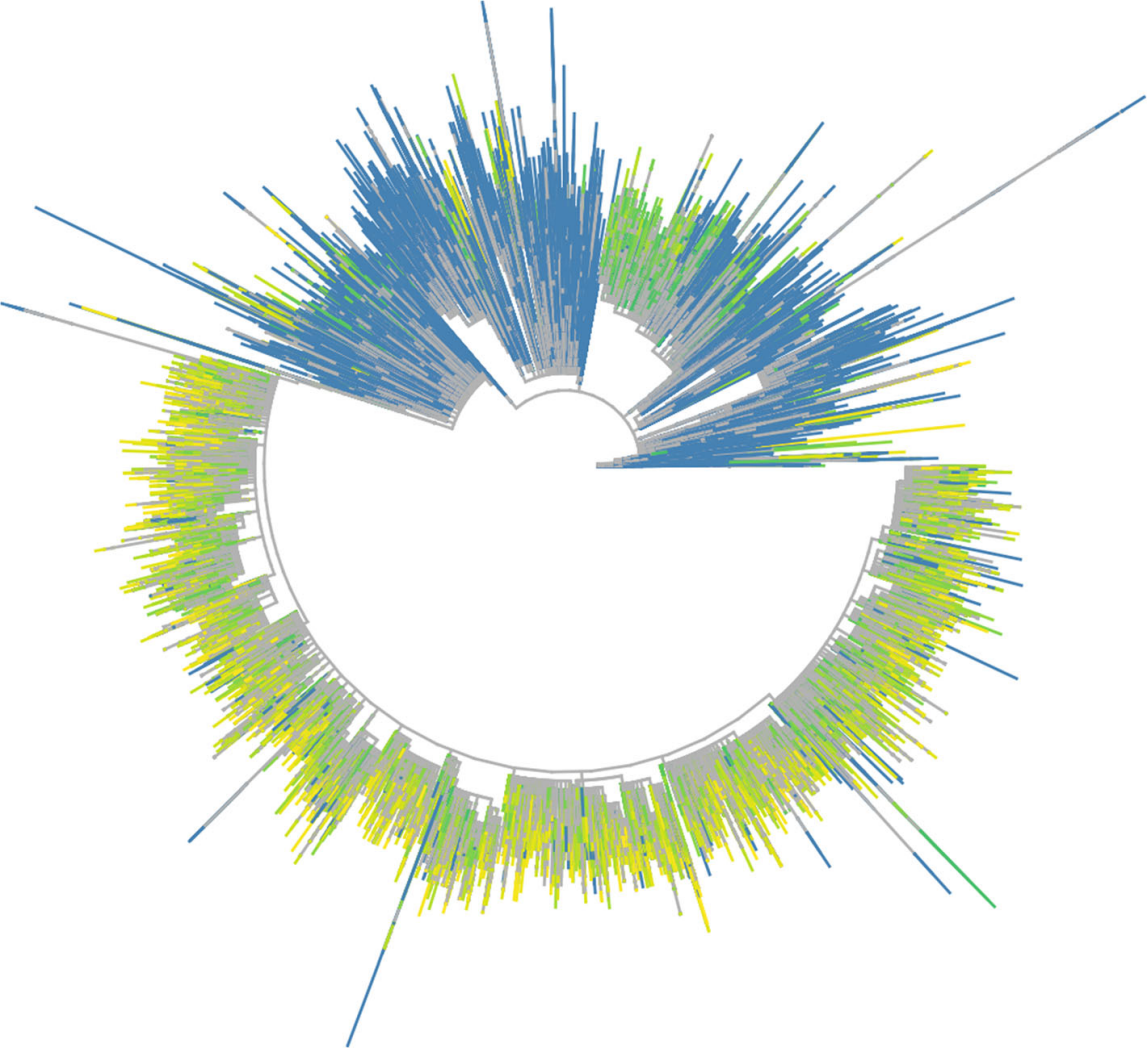
Example tree from Grapevine. A phylogenetic tree of COG-UK sequences (coloured) against a background subset of non-UK sequences from GISAID (grey) from the last 100 days, produced by Grapevine. All UK sequences are coloured by the week in which they were sampled

Elan also triggers the Grapevine phylogenetics pipeline (https://github.com/COG-UK/grapevine). Grapevine is a Snakemake pipeline [13] used to build a phylogenetic tree that captures the evolutionary relationships between the sampled viruses, placing UK sequences in the global context (Fig. 9). Metadata is updated with phylogenetically inferred metrics and both the tree and metadata are made available to the consortium and via CLIMB-COVID’s S3 object store.

As the size of the input data has increased, the phylogenetics pipeline has had to adapt. In January 2021, sequences were filtered by collection date for tree construction, initially including sequences from the most recent 6 months and more recently restricting to the last 100 days. To cope with the scale, a new phylogenetics pipeline (Phylopipe, https://github.com/cov-ert/phylopipe) is under active development. To make the tree building tractable, Phylopipe first performs diversity-aware downsampling of sequences before tree building, then attempts to place excluded sequences back into the tree with UShER [14].

#### Cluster investigations using civet

The global tree, associated metadata and cleaned alignment produced by Grapevine are processed using the Cluster Investigation and Virus Epidemiology Tool (Civet, https://github.com/artic-network/civet). Civet is written in Python and uses Snakemake [13] to orchestrate its analysis steps. Civet allows users to summarise the global and UK-wide diversity of SARS-CoV-2 into interpretable information relevant to their investigation.

**Fig. 10:**
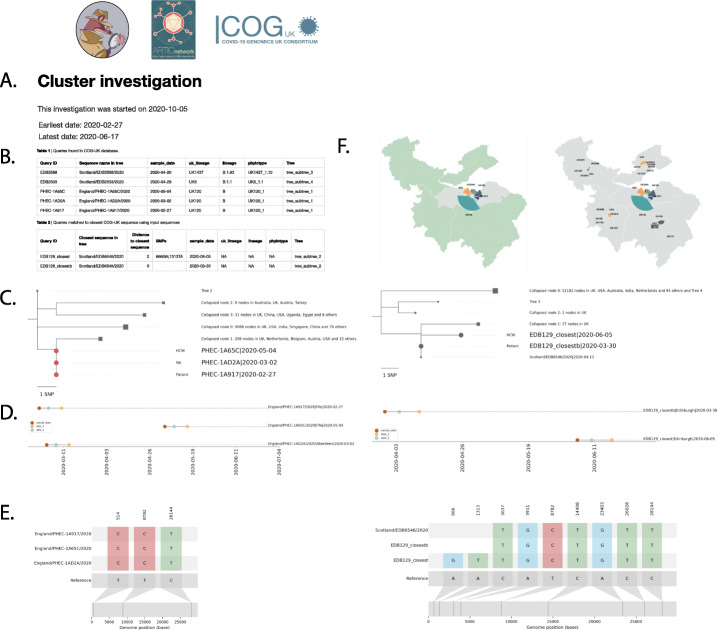
Example Civet report based on a simulated outbreak. The customisable preamble can contain information such as a description of the outbreak, number of sequences of interest and authors of the report (**A**). The report also includes summary tables of the input data, split by whether the queries are in the COG-UK dataset or have been provided by the user (**B**). Civet displays summarised subtrees of the local phylogenetic diversity surrounding sequences of interest, with tips coloured by administrative level one region by default (**C**). Optionally, a timeline of the sequences can be displayed (**D**), and a ‘Snipit graph’ which highlights nucleotide changes from a defined reference genome sequence amongst the sequences of interest (**E**). Descriptive maps show the geographic distribution and the genetic diversity of SARS-CoV-2 circulating in the local area (**F**)

Users can query the dataset using sample COG IDs, a FASTA file of sequences (that may not yet passed through Elan), or query more broadly with criteria such as date and location. Civet produces a customisable report containing summaries of the local phylogenetic diversity between the sequences of interest, as well as figures describing the genetic, temporal and spatial context of the samples (Fig. 10).

#### Linking and visualising consortium data with Microreact

**Fig. 11:**
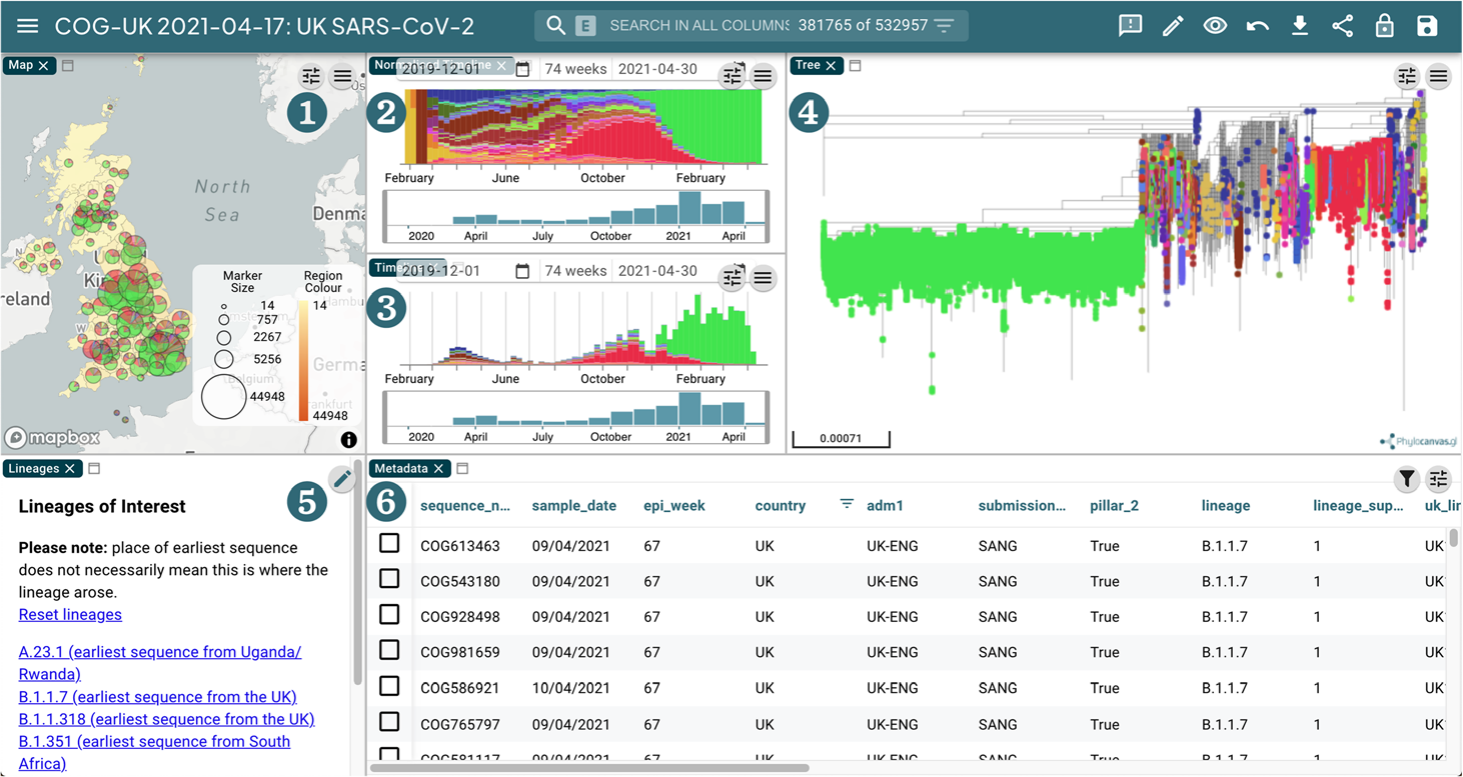
Screenshot of the COG-UK Microreact instance. **1** A map view showing the place of sample collection. **2**, **3** Normalised and standard timelines showing the proportion and number of each lineage found in the samples sequenced over time respectively. **4** A phylogeny derived from the analysis described in the section above. **5** Panel allowing quick filtering by lineages of interest. **6** A metadata table view allowing filtering and sorting of data. These views are generated with COG-UK data that has been processed by Elan and the phylogenetics pipeline

Microreact is a web application that facilitates interpretation of biological data by presenting linked data within a single interactive view [15]. For COG-UK, coarse location metadata from is cleaned and geocoded by analysts, and locations are linked to aesthetic labels with Data-flo (https://data-flo.io). Data-flo provides the ability to manipulate data programmatically and reproducibly using declarative data flows consisting of modular adaptors that perform discrete steps in the overall transformation. The location metadata is combined with the Newick phylogeny from the phylogenetics pipeline to output the COG-UK Microreact instance (Fig. 11), which includes both the COG-UK data and worldwide data from GISAID (https://microreact.org/project/cogconsortium).

Microreact enables querying the data in a visual way that can help inform public health intervention and scientific hypothesis generation. For example, selecting a monophyletic group of genetically very similar samples will update the map and timeline and demonstrate if these samples are co-located in time and space and therefore represent a putative outbreak or transmission chain. The tree viewer is capable of scalable rendering of hundreds of thousands of leaves using Phylocanvas, the WebGL tree viewer developed by the Centre for Genomic Pathogen Surveillance.

#### Distributing sequences and metadata outside the consortium

An important goal for the consortium is to provide other projects and scientists outside of COG-UK access to the sequences and limited metadata to be able to perform analysis of their own. This poses another interoperability problem, as sequences and metadata must be converted into a format acceptable by external databases in order to be deposited.

We archive the raw sequencing reads in the European Nucleotide Archive (ENA) using our pyENA (https://github.com/SamStudio8/pyena) command line client. ENA makes the data available internationally through the International Nucleotide Sequence Database Collaboration (INSDC). Making raw reads available is an important step for external researchers to be able to corroborate findings as well as analyse properties of the reads that are lost when only the consensus genomes are available. Our ENA submission pipeline takes care to mitigate the risk of inadvertently sharing human data by using the Dehumanizer tool (https://github.com/SamStudio8/dehumanizer).

For consensus sequences, the pre-existing usage of GISAID (the Global Initiative on Sharing All Influenza Data [16]) amongst public health laboratories meant that it quickly gained traction as the de facto database to deposit SARS-CoV-2 sequence data. There has been some debate in the wider scientific community about the openness of GISAID and the rules around data access and use; however, within the global health community, GISAID is a trusted route for sharing data. We use the Ocarina command line client to request a subset of the metadata from Majora and automatically generate a suitable CSV and corresponding FASTA file and deposit them daily through the recently released GISAID API client.

We recently developed a mechanism to automate submission of consensus sequences to the INSDC via EMBL-EBI, leveraging the ENA webin client (https://github.com/SamStudio8/elan-ena-nextflow).

## Discussion and conclusion

We have described the end-to-end compute infrastructure we developed for the COVID-19 Genomics UK (COG-UK) consortium. Our platform addresses the needs of a distributed democratised network for sequencing SARS-CoV-2 genomes, providing a unified interface for transferring, storing and sharing sequences and metadata. New metadata is constantly integrated through the Majora API, and downstream sequence and tree datasets are frequently rebuilt by automated pipelines. CLIMB-COVID provides a platform for harmonisation and continuous integration of uploaded sequence and metadata which has underpinned the activities of COG-UK, enabling analysis of over half a million SARS-CoV-2 genomes since its inception.

The funding of CLIMB was a prudent investment, setting the scene for the compute and personnel to be readily available to establish CLIMB-COVID so quickly. CLIMB is probably still the largest dedicated compute infrastructure for microbial genomics in the world. The shared nature of the platform was critical for immediate sharing and analysis across the four nations in the UK. Within 3 days of booting the first virtual machine, we were receiving uploads of sequence data. Within a week, 260 complete genomes from 7 sequencing centres had been uploaded and processed by our inbound distribution pipeline—already more genomes than any other country in the world other than China at the time. Within 2 months, COG-UK was responsible for half of all the international SARS-CoV-2 sequences deposited into GISAID.

Although Black et al. [7] recently suggested it “would be easier to licence databasing software for the metadata database than to build it from scratch”, we had the expertise in place to rapidly develop appropriate software that was unlike anything on the market. Architecting our own database has allowed the metadata definitions, metadata templates and database to evolve together with the changing demands of the consortium. This was especially important given the diverse array of wet and dry laboratory protocols used across the consortium. Our work focussed on building a minimal viable product to address the current needs of the consortium, and then building incremental improvements. This agile methodology allowed us to move quickly, but it does not mean we compromised on functionality: our platform has been built from the ground up by people with domain knowledge. The success of this system speaks to the close working relationship between analysis teams, sample laboratories, the template authors, the authors of the uploading tool and the author of the Majora API. Pipeline developers formed a working group (github.com/COG-UK/dipi-group) to agree, set and communicate standards for transferring data and messages between pipelines and maintain a centralised issue tracker and a log of notable changes to CLIMB-COVID software. If we were given the opportunity to start over, we would make many of the same design choices again.

In our model, data generation and metadata collection are federated across the consortium, but storage and dissemination of data is centralised. This blended model allows us to flexibly support organisations across the country to generate data in a way that leverages their local expertise while offering a single trusted point to immediately validate, access and analyse that data. Our API centred data exchange model has enabled metadata collection and analysis queries to scale to the order of hundreds of thousands of samples.

The availability of single, unique, shareable identifiers across a geographically and organisationally dispersed consortium has been one of the largest obstacles to our work. Our difficulties in obtaining sample and anonymised patient identifiers made it more difficult to link genome sequences to infected people and collate multiple samples from the same individual. Delays in security assessments and contractual arrangements for using granular geographic data left analysts with the unfortunate task of munging various different representations of counties and cities within the UK, and made it more difficult to usefully interrogate phylogenetic data. These metadata issues highlight a need for future readiness, not just for technical solutions, but regulatory ones too. To be ready for the next pandemic, we need a standard methodology for generating shareable identifiers and sharing data between public health agencies, hospital trusts, public and private laboratories backed by a legal framework and capable technical infrastructure.

Establishing the principle of automated and rapid data sharing early on in pandemic response has meant that the UK has become a reliable source of surveillance data and relied upon by other countries to track SARS-CoV-2 lineage dynamics. Established early as part of a surveillance protocol, such a model helps prevent data sharing being latterly suppressed by concerns around political ramifications of data sharing such as sensitivities around border policy.

**Fig. 12:**
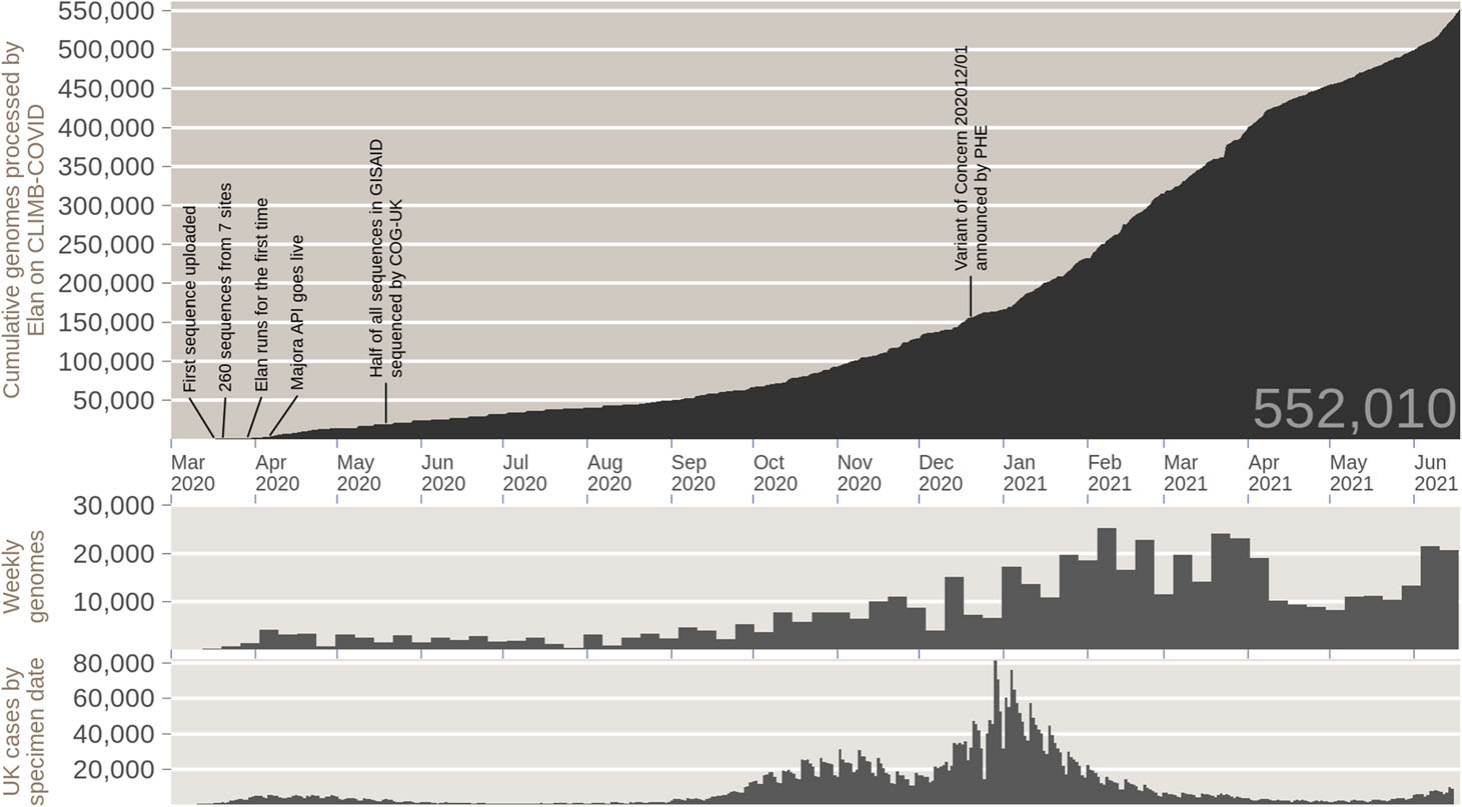
Cumulative total sequences and weekly sequences processed by Elan on CLIMB-COVID, compared to UK daily SARS-CoV-2 positive cases. (Top) Cumulative total genomes processed by Elan on CLIMB-COVID since March 2020. (Middle) Number of genomes processed per week by Elan on CLIMB-COVID. (Bottom) Daily positive SARS-CoV-2 cases in the UK (by specimen date) as reported by the UK government website for data and insights on coronavirus (coronavirus.data.gov.uk)

The infrastructure we have presented here is generalizable to future novel pathogens, but could also be expanded to cover metagenomics and environmental sampling. CLIMB-COVID is a proven model, evidenced by the success of the COG-UK consortium (Fig. 12). As of writing, COG-UK has produced over 550,000 public sequences, has contributed more than 20 reports to the government and 50 academic publications and supported hundreds of outbreak investigations across the UK. CLIMB-COVID has enabled high profile analyses including within-host diversity of SARS-CoV-2 [17], the effects of SARS-CoV-2 Spike Mutation D614G on transmissibility and pathogenicity [18] and lineage dynamics of the SARS-CoV-2 epidemic in the UK [19]. COG-UK was instrumental in the identification of the SARS-CoV-2 B.1.1.7 lineage in December 2020 [20], which was Public Health England’s first designated variant of concern (VOC 202012/01) [21].

Our efforts have enabled us to go from a blank slate to an integrated infrastructure that coalesces the sequence and metadata from multiple sequencing centres spread across four distinct healthcare systems. The model we present here should be an example for those who have similar objectives, as well as presenting a very different vision to those who would suggest that data should be centralised into databases that sit apart from analysis tools and detailed medata.

## Supplementary Information

**Additional file 1.** The COVID-19 Genomics UK (COG-UK) Consortium.

**Additional file 2: Table 3.** COG-UK full metadata standard.

## Data Availability

Majora, the Django web application for tracking artifacts and processes, is open source and freely distributed under the MIT license via github.com/SamStudio8/majora. The Ocarina command line client reference implementation for using the Majora API is open source and freely distributed under the MIT license via github.com/SamStudio8/ocarina. The Elan inbound distribution Nextflow pipeline is open source and freely distributed under the MIT license via github.com/SamStudio8/elan-nextflow. The Swell programme to calculate QC metrics from BAM depth files is open source and freely distributed under the MIT license via github.com/SamStudio8/swell. The Dehumanizer programme to sanitise BAMs is open source and freely distributed under the MIT license via github.com/SamStudio8/dehumanizer. The PyENA programme to upload BAMs to ENA is open source and freely distributed under the MIT license via github.com/SamStudio8/pyena. The Elan outbound distribution Nextflow pipeline for submitting consensus sequences to ENA/INSDC is open source and freely distributed under the MIT license via github.com/SamStudio8/elan-ena-nextflow. The ARTIC fieldbioinformatics toolkit for working with viral nanopore sequencing data is open source and freely distributed under the MIT license via github.com/artic-network/fieldbioinformatics. The Nextflow pipeline for automating the ARTIC nCoV-2019 bioinformatics protocol is open source and freely distributed under the AGPL-3.0 license via github.com/connor-lab/ncov2019-artic-nf. Grapevine, the Snakemake pipeline for processing consensus sequences and conducting phylogenetic tree building, is open source and freely distributed under the GPL-3.0 License via github.com/COG-UK/grapevine. The Datapipe Nextflow pipeline for post-processing of the COG-UK dataset and PANGO lineage assignment is open source and freely distributed under the GPL-3.0 License via github.com/COG-UK/datapipe. Phylopipe, the second phylogenetics pipeline for diversity-aware downsampling of sequences and UShER backfilling, is open source and freely distributed under the GPL-3.0 License via github.com/cov-ert/phylopipe. Civet, the Snakemake-based tool for generating interpretable reports from phylogenetic data, is open source and freely distributed under the GPL-3.0 License via github.com/artic-network/civet. Snipit, for generating Civet figures that show SNPs relative to a reference sequence, is open source and freely distributed under the GPL-3.0 License via github.com/aineniamh/snipit. Consensus sequences, alignments, metadata and trees are publicly hosted on CLIMB-COVID S3 with links available via data.covid19.climb.ac.uk. Consensus SARS-CoV-2 genomes are routinely deposited into GISAID. Consensus SARS-CoV-2 genomes and human-filtered sequencing data are routinely deposited in the European Nucleotide Archive (ENA) at EMBL-EBI under accession PRJEB37886. COG-UK data can be explored using a Microreact instance available at microreact.org/project/cogconsortium.
