# Supplementary material for: CLIMB-COVID: continuous integration supporting decentralised sequencing for SARS-CoV-2 genomic surveillance
Source: Genome Biol. 2021 Jul 1;22:196. doi: 10.1186/s13059-021-02395-y (PMC8247108; doi:10.1186/s13059-021-02395-y)
Supplement: Supplementary file 2 — Additional file 2: Table 3. COG-UK full metadata standard. [file 13059_2021_2395_MOESM2_ESM.docx]

# Supplementary information

## Full COG-UK metadata specification

| \| **Data item** \| **Field name** \| **Description** \| **Access level**  **(Public, Consortium or Restricted)** \| **Mandatory** \| **Implementation** \| \| --- \| --- \| --- \| --- \| --- \| --- \| \| **Sample identifiers** \| \| \| \| \| \| \| **Central Sample ID** \| central_sample_id \| A unique identifier to refer to the sample within the consortium \| Public \| Yes \| Tier 1 database model (biosample) \| \| **COG-UK Patient ID** \| biosample_source_id \| A unique identifier to refer to the person who was sampled within the consortium \| Consortium \| No \| Tier 1 database model (biosample source) \| \| **PHA sample ID** \| root_sample_id \| If available, the identifier assigned to the sample by one of the four public health agencies \| Restricted \| No \| Tier 1 database model (biosample) \| \| **Local sample ID** \| sender_sample_id \| The identifier used to refer to this sample at the laboratory that submitted the sample to the consortium \| Restricted \| No \| Tier 1 database model (biosample) \| \| **Sample details** \| \| \| \| \| \| \| **Date of sample**  **(collected)** \| collection_date \| The date the sample was collected \| Public \| Yes (otherwise received_date) \| Tier 1 database model (biosample collection) \| \| **Date of sample (received)** \| received_date \| The earliest date that this sample was known to be checked in to a diagnostic or sequencing laboratory \| Public \| No (unless collection_date is not provided) \| Tier 1 database model (biosample collection) \| \| **Collecting organisation** \| collecting_org \| The full name of the organisation that collected the sample \| Consortium \| No \| Tier 1 database model (biosample collection) \| \| **Sampling strategy** \| is_surveillance \| Whether this sample was collected as part of a random surveillance strategy, or a targeted outbreak analysis \| Consortium \| Yes \| Tier 2 database model (COG-UK biosample collection supplement) \| \| **Sample type collected** \| sample_type_collected \| Type of sample at collection \| Consortium \| No \| Tier 1 database model (biosample collection) \| \| **Swab site** \| swab_site \| Site of swab \| Consortium \| No \| Tier 1 database model (biosample collection) \| \| **Sample type received** \| sample_type_received \| Type of sample received by sequencing lab \| Consortium \| No \| Tier 1 database model (biosample collection) \| \| **Ct value** \| ct_N_ct_value \| Observed cycle threshold value for Ct test N \| Consortium \| No \| Tier 2 database model (cycle threshold metric) \| \| **Ct test kit** \| ct_N_test_kit \| Kit used to prepare Ct test N \| Consortium \| No \| Tier 2 database model (cycle threshold metric) \| \| **Ct test platform** \| ct_N_test_platform \| Platform used to conduct Ct test N \| Consortium \| No \| Tier 2 database model (cycle threshold metric) \| \| **Ct test target** \| ct_N_test_target \| Gene target of Ct test N \| Consortium \| No \| Tier 2 database model (cycle threshold metric) \| \| **Demographics and employment** \| \| \| \| \| \| \| **Country code** \| adm1 \| The country in which the sample was collected \| Public \| Yes \| Tier 1 database model (biosample collection) \| \| **County** \| adm2 \| The county within the United Kingdom in which the sample was collected \| Consortium \| Strongly recommended \| Tier 1 database model (biosample collection) \| \| **Outer postcode** \| adm2_private \| The outer postcode of the home address for the sampled person \| Consortium \| No \| Tier 1 database model (biosample collection) \| \| **Age** \| source_age \| Patient age \| Consortium \| No \| Tier 1 database model (biosample collection) \| \| **Sex** \| source_sex \| Patient sex \| Consortium \| No \| Tier 1 database model (biosample collection) \| \| **Healthcare worker** \| is_hcw \| Is the sample from a health care worker? \| Restricted \| No \| Tier 2 database model (COG-UK biosample collection supplement) \| \| **Employing hospital** \| employing_hospital_name \| Name of the hospital if a health care worker who works in a hospital. \| Restricted \| No \| Tier 2 database model (COG-UK biosample collection supplement) \| \| **Employing trust / board** \| employing_hospital_trust_or_board \| Name of the trust / health board if a health care worker who works in a hospital. \| Restricted \| No \| Tier 2 database model (COG-UK biosample collection supplement) \| \| **Care home worker** \| is_care_home_worker \| Is the sample from a care home worker? \| Restricted \| No \| Tier 2 database model (COG-UK biosample collection supplement) \| \| **Care home resident** \| is_care_home_resident \| Is the sample from a care home resident? \| Restricted \| No \| Tier 2 database model (COG-UK biosample collection supplement) \| \| **Care home ID** \| anonymised_care_home_code \| Locally assigned anonymous code (up to ten characters) that links samples from the same care home. \| Restricted \| No \| Tier 2 database model (COG-UK biosample collection supplement) \| \| **Case severity and outcome** \| \| \| \| \| \| \| **Hospitalisation** \| is_hospital_patient \| Is the sample from an admitted hospital patient? \| Restricted \| No \| Tier 2 database model (COG-UK biosample collection supplement) \| \| **Date of admission** \| admitted_date \| Date of admission to hospital \| Restricted \| No \| Tier 2 database model (COG-UK biosample collection supplement) \| \| **Admitting hospital** \| admitted_hospital_name \| Name of the hospital if a hospital patient. \| Restricted \| No \| Tier 2 database model (COG-UK biosample collection supplement) \| \| **Admitting trust or board** \| admitted_hospital_trust_or_board \| Name of the trust / health board if a hospital patient. \| Restricted \| No \| Tier 2 database model (COG-UK biosample collection supplement) \| \| **Admitted with COVID-19 diagnosis** \| admitted_with_covid_diagnosis \| Was the patient admitted with a (suspected) diagnosis of COVID-19? \| Restricted \| No \| Tier 2 database model (COG-UK biosample collection supplement) \| \| **Critical care admission** \| is_icu_patient \| Whether patient has been admitted to ICU before sample collection \| Restricted \| No \| Tier 2 database model (COG-UK biosample collection supplement) \| \| **Sequencing Library** \| \| \| \| \| \| \| **Library name** \| library_name \| A unique name for the library. \| Public \| Yes \| Tier 1 database model (library) \| \| **Library layout** \| library_layout_config \| Whether the run has single or paired reads \| Public \| Yes \| Tier 1 database model (library) \| \| **Sequencing kit** \| library_seq_kit \| The sequencing kit used \| Public \| No \| Tier 1 database model (library) \| \| **Sequencing protocol** \| library_seq_protocol \| The sequencing protocol used \| Public \| No \| Tier 1 database model (library) \| \| **Insert length** \| library_layout_insert_length \| Nominal length of sequencing library insert. (Illumina only) \| Public \| No \| Tier 1 database model (library) \| \| **Read length** \| library_layout_read_length \| Nominal length of sequencing library reads (Illumina only) \| Public \| No \| Tier 1 database model (library) \| \| **Library Central Sample ID** \| biosample_N_central_sample_id \| Central sample ID for biosample N included in the library \| Public \| Yes \| Tier 2 database model (library record) \| \| **Library selection** \| biosample_N_library_selection \| Selection / enrichment method for biosample N included in the library \| Public \| Yes \| Tier 2 database model (library record) \| \| **Library source** \| biosample_N_library_source \| Source material for biosample N included in the library \| Public \| Yes \| Tier 2 database model (library record) \| \| **Library strategy** \| biosample_N_library_strategy \| Library preparation strategy for biosample N included in the library \| Public \| Yes \| Tier 2 database model (library record) \| \| **Primer version** \| biosample_N_library_primers \| The version of the primers used for biosample N included in the library \| Public \| No \| Tier 2 database model (library record) \| \| **Barcode** \| biosample_N_barcode \| Barcode adaptor/number for biosample N included in the library \| Public \| No \| Tier 2 database model (library record) \| \| **Sequencing run** \| \| \| \| \| \| \| **Run name** \| run_name \| A unique name for the run \| Public \| Yes \| Tier 1 database model (sequencing run) \| \| **Instrument make** \| instrument_make \| Make of instrument used \| Public \| Yes \| Tier 1 database model (sequencing run) \| \| **Instrument model** \| instrument_model \| Model of instrument used \| Public \| Yes \| Tier 1 database model (sequencing run) \| \| **Flowcell ID** \| flowcell_id \| Flowcell serial number \| Public \| No \| Tier 1 database model (sequencing run) \| \| **Flowcell type** \| flowcell_type \| Flowcell description \| Public \| No \| Tier 1 database model (sequencing run) \| \| **Start time** \| start_time \| Start time of run \| Public \| No \| Tier 1 database model (sequencing run) \| \| **End time** \| end_time \| End time of run \| Public \| No \| Tier 1 database model (sequencing run) \| |
| --- | --- | --- | --- | --- | --- | --- | --- | --- | --- | --- | --- | --- | --- | --- | --- | --- | --- | --- | --- | --- | --- | --- | --- | --- | --- | --- | --- | --- | --- | --- | --- | --- | --- | --- | --- | --- | --- | --- | --- | --- | --- | --- | --- | --- | --- | --- | --- | --- | --- | --- | --- | --- | --- | --- | --- | --- | --- | --- | --- | --- | --- | --- | --- | --- | --- | --- | --- | --- | --- | --- | --- | --- | --- | --- | --- | --- | --- | --- | --- | --- | --- | --- | --- | --- | --- | --- | --- | --- | --- | --- | --- | --- | --- | --- | --- | --- | --- | --- | --- | --- | --- | --- | --- | --- | --- | --- | --- | --- | --- | --- | --- | --- | --- | --- | --- | --- | --- | --- | --- | --- | --- | --- | --- | --- | --- | --- | --- | --- | --- | --- | --- | --- | --- | --- | --- | --- | --- | --- | --- | --- | --- | --- | --- | --- | --- | --- | --- | --- | --- | --- | --- | --- | --- | --- | --- | --- | --- | --- | --- | --- | --- | --- | --- | --- | --- | --- | --- | --- | --- | --- | --- | --- | --- | --- | --- | --- | --- | --- | --- | --- | --- | --- | --- | --- | --- | --- | --- | --- | --- | --- | --- | --- | --- | --- | --- | --- | --- | --- | --- | --- | --- | --- | --- | --- | --- | --- | --- | --- | --- | --- | --- | --- | --- | --- | --- | --- | --- | --- | --- | --- | --- | --- | --- | --- | --- | --- | --- | --- | --- | --- | --- | --- | --- | --- | --- | --- | --- | --- | --- | --- | --- | --- | --- | --- | --- | --- | --- | --- | --- | --- | --- | --- | --- | --- | --- | --- | --- | --- | --- | --- | --- | --- | --- | --- | --- | --- | --- | --- | --- | --- | --- | --- | --- | --- | --- | --- | --- | --- | --- | --- | --- | --- | --- | --- | --- | --- | --- | --- | --- | --- | --- | --- | --- | --- | --- | --- | --- | --- | --- | --- | --- | --- | --- | --- | --- | --- | --- | --- | --- | --- | --- | --- | --- | --- | --- | --- | --- | --- | --- | --- | --- | --- | --- | --- | --- | --- | --- | --- | --- | --- | --- | --- | --- | --- | --- | --- | --- | --- | --- | --- | --- | --- | --- | --- | --- | --- | --- | --- |
| [Table 3](#table_metadatafull) **COG-UK full metadata standard** |

## 
